# Cannabis, Intraocular Pressure, and the Growth Arrest-Specific 7 (GAS7) Gene: A Retrospective Analysis

**DOI:** 10.7759/cureus.23919

**Published:** 2022-04-07

**Authors:** Steven Lehrer, Peter H Rheinstein

**Affiliations:** 1 Radiation Oncology, Icahn School of Medicine at Mount Sinai, New York, USA; 2 Family Medicine, Severn Health Solutions, Severna Park, USA

**Keywords:** gas7, gene, therapy, glaucoma, cannabis

## Abstract

Background

Intraocular pressure (IOP) is a highly heritable risk factor for primary open-angle glaucoma (POAG), with at least 27 related genes; however, we are still not aware as to which receptors or genes that the main components of cannabis use to lower IOP.

Methods

In the current study, we used data from the UK Biobank (UKBB) to assess the relationship of growth arrest-specific 7 (GAS7) with IOP and cannabis in 37,046 subjects. GAS7, at chromosome 17p31.1, is quite close to a cannabis receptor at chromosome 17p31.3. For comparison, we chose a second IOP/glaucoma gene, CDKN2B-AS1 on chromosome 9p21.3, with no known relationship to cannabis. In addition, we examined the effect of CB1, GPR18, and cannabis on IOP; these two genes are associated with cannabis IOP reduction in mice.

Results

Total cannabis use versus IOP and genotypes of GAS7 SNP rs9913911 in the 37,046 subjects showed significant variation [p<0.001, univariate analysis of variance (ANOVA)]. Carriers of the GAS7 rs9913911 minor allele G had lower IOP with increased cannabis use. Total cannabis use versus IOP of genotypes of CDKN2B-AS1 SNP rs944801 in 37,046 subjects had IOP variability with cannabis use that was insignificant (p=0.138). We analyzed the relationship of CB1 SNP rs806365 and GPR18 SNP rs3742130 with cannabis use and IOP, which was insignificant. CB1 and GPR18 are probably not involved in cannabis-associated human IOP reduction, unlike what has been reported in mice.

Conclusion

Cannabis-based treatments, which apparently act on the GAS7 gene, can be utilized to reduce IOP. However, their disadvantages outweigh their advantages, which was not the case when the initial reports of marijuana's effects on IOP were published in the 1970s. Hence, cannabis-based glaucoma treatments are now of questionable value.

## Introduction

Cannabis has a long and colorful history stretching back thousands of years. The main psychotropic element in cannabis is thought to be 9-tetrahydrocannabinol (THC). In 1971, Hepler and Frank published their first study that established that cannabis inhalation reduces intraocular pressure (IOP) [1]. IOP is a highly heritable risk factor for primary open-angle glaucoma (POAG), with at least 27 related genes [2], but we still do not know which genes or receptors the main components of cannabis use to lower IOP.

CB1 receptors are the best-characterized cannabinoid receptors. Because cannabinoid CB1 receptor agonists lower IOP in mice, THC might work via CB1 receptors, which are widely distributed in the brain and eyes. CB1 receptors govern critical physiological systems such as pain, emotion, locomotion, and memory. The cannabinoid signaling system contains other receptors, such as GPR18. In a study of mice, Miller et al. found that THC lowers IOP by activating CB1 and GPR18 in a sex-dependent manner [3].

Growth arrest-specific 7 (GAS7), one of the genes that influence IOP, is found in a chromosomal area previously identified by a glaucoma linkage study and subsequently by a genome-wide association study (GWAS) [4]. GAS7, at chromosome 17p31.1, is quite close to a cannabis receptor at chromosome 17p31.3 [5] and has been associated with schizophrenia in a Chinese population [6].

In the current study, we used data from the UK Biobank (UKBB) to assess the relationship of GAS7 with IOP and cannabis. For comparison, we chose a second IOP/glaucoma gene, CDKN2B-AS1 on chromosome 9p21.3 [7], with no known relationship to cannabis. In addition, we examined the effect of CB1, GPR18, and cannabis on IOP.

## Materials and methods

The UKBB is a large prospective observational study of men and women. Participants were recruited from across 22 centers located throughout England, Wales, and Scotland between 2006 and 2010 and continue to be longitudinally followed for the capture of subsequent health events [8]. This methodology is similar to that of the ongoing Framingham Heart Study [9], with the exception that the UKBB program collects postmortem samples, which Framingham did not.

Our UKBB application was approved as UKB project 57245 (S.L., P.H.R.). Our analysis included all subjects with glaucoma and cannabis-smoking information. POAG diagnosis was ascertained using the 10th Revision of the International Classification of Diseases (ICD-10), H40.1. The age when glaucoma was diagnosed was collected from data field 4700. Cannabis information was recorded in the UKB category 143, data field 20453, ever taken cannabis. A touch screen posed the question, "Have you taken CANNABIS (cannabis, grass, hash, ganja, blow, draw, skunk, weed, spliff, dope), even if it was a long time ago?" Answers available were no; yes, 1-2 times; yes, 3-10 times; yes, 11-100 times; yes, more than 100 times.

Data processing was performed on Minerva, a Linux mainframe with Centos 7.6, at the Icahn School of Medicine at Mount Sinai. We used PLINK, a whole-genome association analysis toolset, to analyze the UKBB chromosome files [10] and the UK Biobank Data Parser (ukbb parser), a python-based package that allows easy interfacing with the large UKBB dataset [11]. Statistical analysis was done with SPSS Statistics version 26 (IBM, Armonk, NY) and R. We used the univariate general linear model of SPSS, which allows analysis of variance (ANOVA) for two fixed factors, in this case, two single-nucleotide polymorphisms (SNPs).

## Results

The mean age of 37,046 subjects was 56 ± 8 years; 54% of them were female, and 46% were male; 95% were white British. Of note, 633 of the subjects had POAG. The four SNPs analyzed are detailed in Table 1. IOP is corneal-compensated for the right eye.

**Table 1:** SNPs analyzed in this study SNP: single-nucleotide polymorphism; MAF: minor allele frequency

Glaucoma gene analyzed	Chromosome location	SNP of glaucoma gene analyzed	Major allele		Minor allele	MAF
GAS7	17p13.1	rs9913911	A	>	G	0.44
CDKN2B-AS1	9p21.3	rs944801	C	>	G	0.41
CB1	6q15	rs806365	C	>	T	0.41
GPR18	13q32.3	rs3742130	G	>	A	0.2

**Figure 1:**
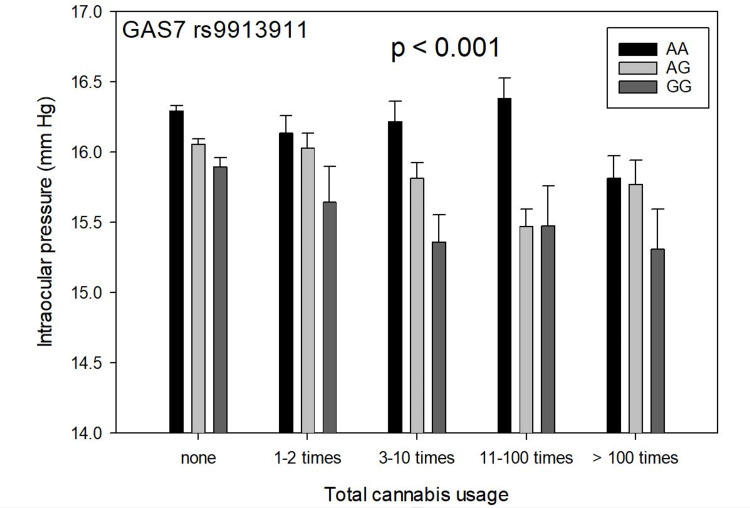
Total cannabis use versus intraocular pressure, mean + SEM, and genotypes of GAS7 SNP rs9913911 in 37,046 subjects (p<0.001, univariate ANOVA) Carriers of the minor allele G had lower IOP with increased cannabis use. Tukey's B post hoc range test showed that carriers of the minor allele G who had used cannabis 11-100 times had significantly lower IOP than the three other groups (p=0.05), whereas subjects who had not used cannabis had significantly higher IOP than the next three groups (p=0.05). There was no significant IOP effect of cannabis use on homozygotes for the major allele A (black bars, p=0.089) SEM: standard error of the mean; GAS7: growth arrest-specific 7; ANOVA: analysis of variance; IOP: intraocular pressure

Figure 1 shows total cannabis use versus IOP (mean + SEM) and genotypes of GAS7 SNP rs9913911 in 37,046 subjects (p<0.001, univariate ANOVA). Carriers of the minor allele G had lower IOP with increased cannabis use. Tukey's B post hoc range test showed that carriers of the minor allele G who had used cannabis 11-100 times had significantly lower IOP than the three other groups (p=0.05), whereas subjects who had not used cannabis had significantly higher IOP than the next three groups (p=0.05). There was no significant IOP effect of marijuana use on homozygotes for the major allele A (p=0.089). 

**Figure 2:**
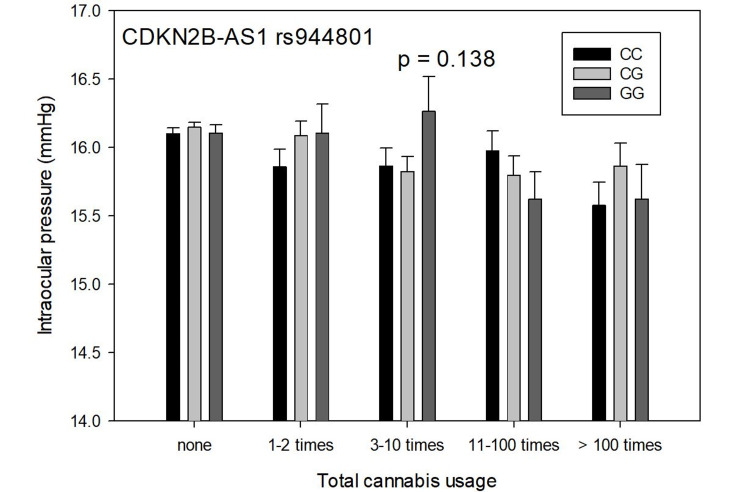
Total cannabis use versus intraocular pressure, mean + SEM, and genotypes of CDKN2B-AS1 SNP rs944801 in 37,046 subjects The IOP variability with cannabis use was insignificant (p=0.138) SEM: standard error of the mean; IOP: intraocular pressure

Figure 2 shows total cannabis use versus IOP, mean + SEM, and genotypes of CDKN2B-AS1 SNP rs944801 in 37,046 subjects. The IOP variability with cannabis use was insignificant (p=0.138).

To test the relationship of rs9913911 and rs7518099 with cannabis use and IOP, an analysis of covariance between rs9913911 and rs7518099 was performed, with age-weighted least-squares regression using the univariate general linear model of SPSS. IOP was the dependent variable, rs9913911 and rs7518099 were fixed factors, and cannabis use and the presence or absence of POAG were covariates. The interaction of rs9913911 and rs7518099 was significant (p=0.014). In other words, the overall results in Figure 1 and Figure 2 were significantly different from each other. IOP varied significantly with the GAS7 rs9913911 genotype when compared to the CDKN2B-AS1 SNP rs944801 genotype.

To test the relationship of CB1 SNP rs806365 and GPR18 SNP rs3742130 with cannabis use and IOP, an analysis of covariance between rs806365 and rs3742130 was performed, with age-weighted least-squares regression using the univariate general linear model of SPSS. IOP was the dependent variable, rs806365 and rs3742130 were fixed factors, and cannabis use and the presence or absence of POAG were covariates. The rs806365 genotype had no significant effect on IOP (p=0.083), and the rs3742130 genotype had no significant effect on IOP (p=0.831) either. The interaction of rs806365 and rs3742130 was not significant (p=0.574). In other words, CB1 and GPR18 are probably not involved in cannabis-associated human IOP reduction, unlike what has been reported in mice [3].

## Discussion

The optic nerve, specifically the lamina cribrosa, has a high level of GAS7 expression. The lamina cribrosa is the connective tissue network that the nerve fibers pass through to create the optic nerve and is likely the primary location of glaucomatous optic nerve injury. The ciliary body (CB), the secretory neuroepithelium that creates aqueous humor, has moderate to high GAS7 expression. The trabecular meshwork (TM), which is the primary tissue involved in aqueous humor outflow [12], has a high GAS7 expression. The CB and TM in tandem mostly control IOP [4]. A cannabis receptor, vanilloid 1 (TRPV1), which is 44-kb long, is at chromosome 17p13.3, quite close to GAS7 at 17p13.1. TRPV1 may serve as a unique receptor site for endogenous, and possibly exogenous, cannabinoids [5]. Thus, the association of GAS7 with IOP and cannabis might be expected.

It is scarcely unexpected that CB1 and GPR18 are implicated in cannabis-induced IOP reduction in mice but not in humans, as the UKBB results imply. Humans, rats, and mice have almost the same number of genes, according to a genome analysis conducted by 20 institutions from six nations. Humans and rodents, on the other hand, descended from a common ancestor 80 million years ago, with rats and mice diverging between 12 and 24 million years ago [13]. CB1 and GPR18 may have evolved vastly different functions in mice and humans over millions of years.

Cannabinoids or marijuana reduce IOP as well as do most standard glaucoma treatments. This is true whether the cannabinoids are taken orally, intravenously, or inhaled, but not when they are directly applied to the eyes. In both glaucoma patients and healthy people with normal IOP, smoked or eaten marijuana, THC and synthetic cannabinoids in pill form, and intravenous injections of many natural cannabinoids all reduce IOP considerably. A single dose of marijuana or cannabinoid has maintained this effect for three to four hours in most trials [14].

However, inhaling cannabis smoke is injurious to the eye. In the case of glaucoma, the manifestations are different from those of inhaled tobacco smoke. Cannabis reduces IOP but accelerates glaucoma development. Cannabis does not increase the risk of glaucoma. Cigarette smoking increases the risk of glaucoma and significantly accelerates glaucoma development. But the early use of cannabis leads to the development of glaucoma earlier in life [15].

Cannabis has other drawbacks as well. It lowers blood pressure and has psychological effects that some individuals, especially the elderly, find unpleasant. After taking cannabis, patients may claim that their hearts pound or race, and that they feel uncomfortably tense. All these effects could be particularly dangerous for patients who are at risk of cardiovascular disease and stroke; also, lower blood pressure may diminish blood supply to the optic nerve, negating the benefits of lower IOP. Finally, because of their short duration of action, cannabis-based drugs must be taken up to eight times per day, which most patients are unlikely to do. Currently available medicines reduce IOP just as well and require only one or two doses per day. This is a significant distinction because, due to the degenerative nature of glaucoma, patients must maintain constant IOP control [14].

Our study has some limitations. The normal IOP is 10-21 mmHg. The difference in means associated with cannabis and the GAS7 rs9913911 alleles represents a small band within this range. It is possible that the observed difference is significant because of the large sample size, but too small to be clinically relevant. Moreover, the cannabinoid cannabigerol is a highly potent alpha2-adrenoceptor agonist [16] and might reduce IOP like the alpha2 agonist brimonidine, a glaucoma medication [17].

## Conclusions

Cannabis-based treatments, which may interact with the GAS7 gene, can be utilized to reduce IOP. However, their disadvantages exceed their advantages, which was not the case when the initial reports of marijuana's effects on IOP were published in the 1970s. Earlier, only a small number of medicines were available to reduce IOP, all of which had serious negative effects. More effective pharmaceuticals with fewer side effects have since supplanted these drugs, and cannabis-based glaucoma treatments are now of questionable value.
